# Supplementary figures and images for: Model-based translation of DNA damage signaling dynamics across cell types
Source: PLoS Comput Biol. 2022 Jul 8;18(7):e1010264. doi: 10.1371/journal.pcbi.1010264 (PMC9269748; doi:10.1371/journal.pcbi.1010264)

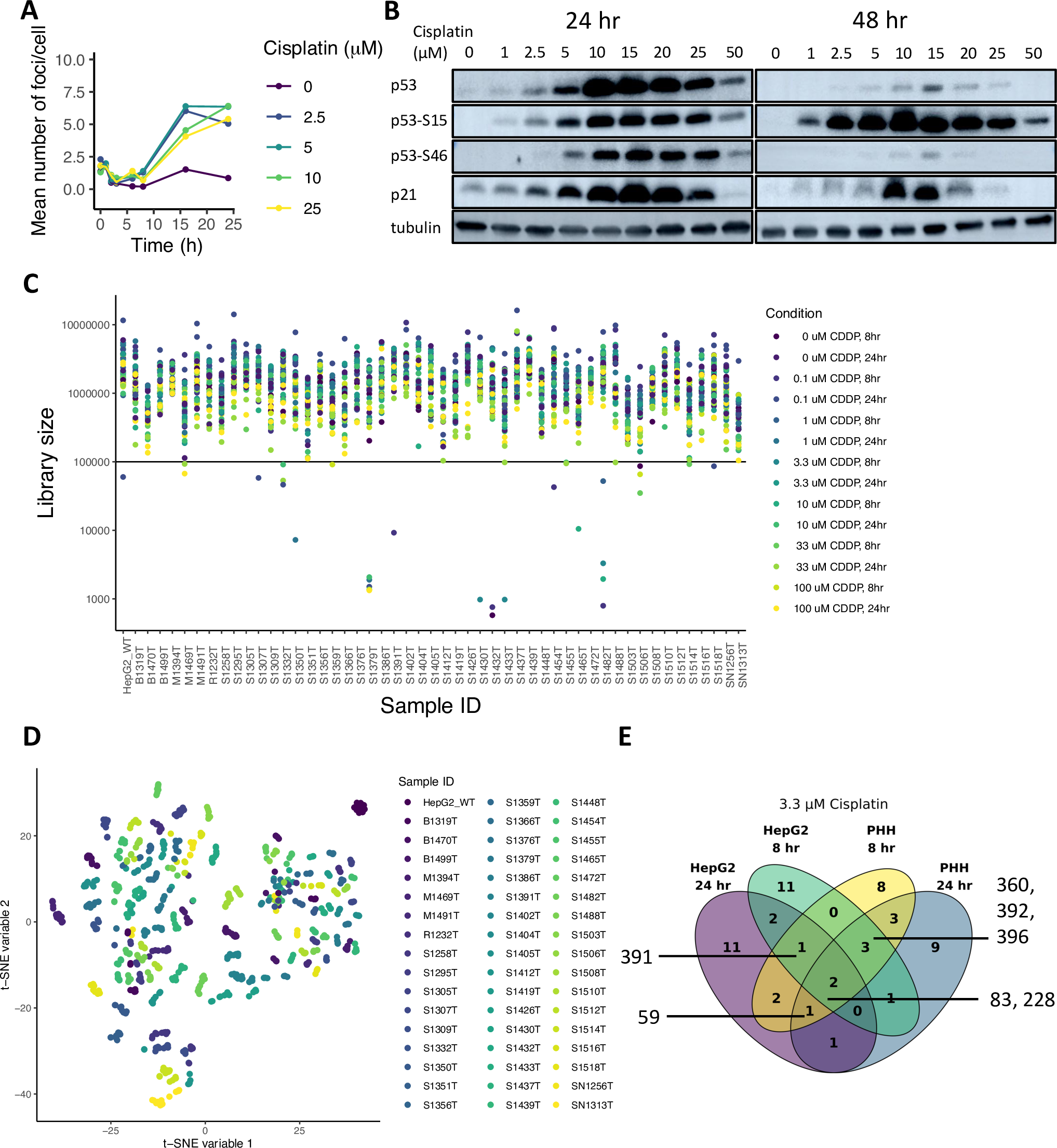

Supplement: S1 Fig — (A) Mean number of γ-H2AX foci in HepG2 cells over time after cisplatin exposure (n = 1). (B) Expression of p53, p53-S15, p53-S46 and p21 at 24 (left) or 48 (right) hours after cisplatin exposure, as measured by Western blot (n = 1). (C) TempO-Seq library sizes per measurement and sample. Conditions with less than 100,000 reads (solid black line) are discarded from the analysis. CDDP, cisplatin. (D) Distribution of samples based on the expression of all genes in the S1500+ gene set after dimensionality reduction with PCA followed by t-SNE. (E) Overlap in the 20 TXG-MAPr modules with the highest Eigengene score per cell type at 8 and 24 hours after exposure to 3.3 μM cisplatin. (TIF) [file pcbi.1010264.s001.tif]

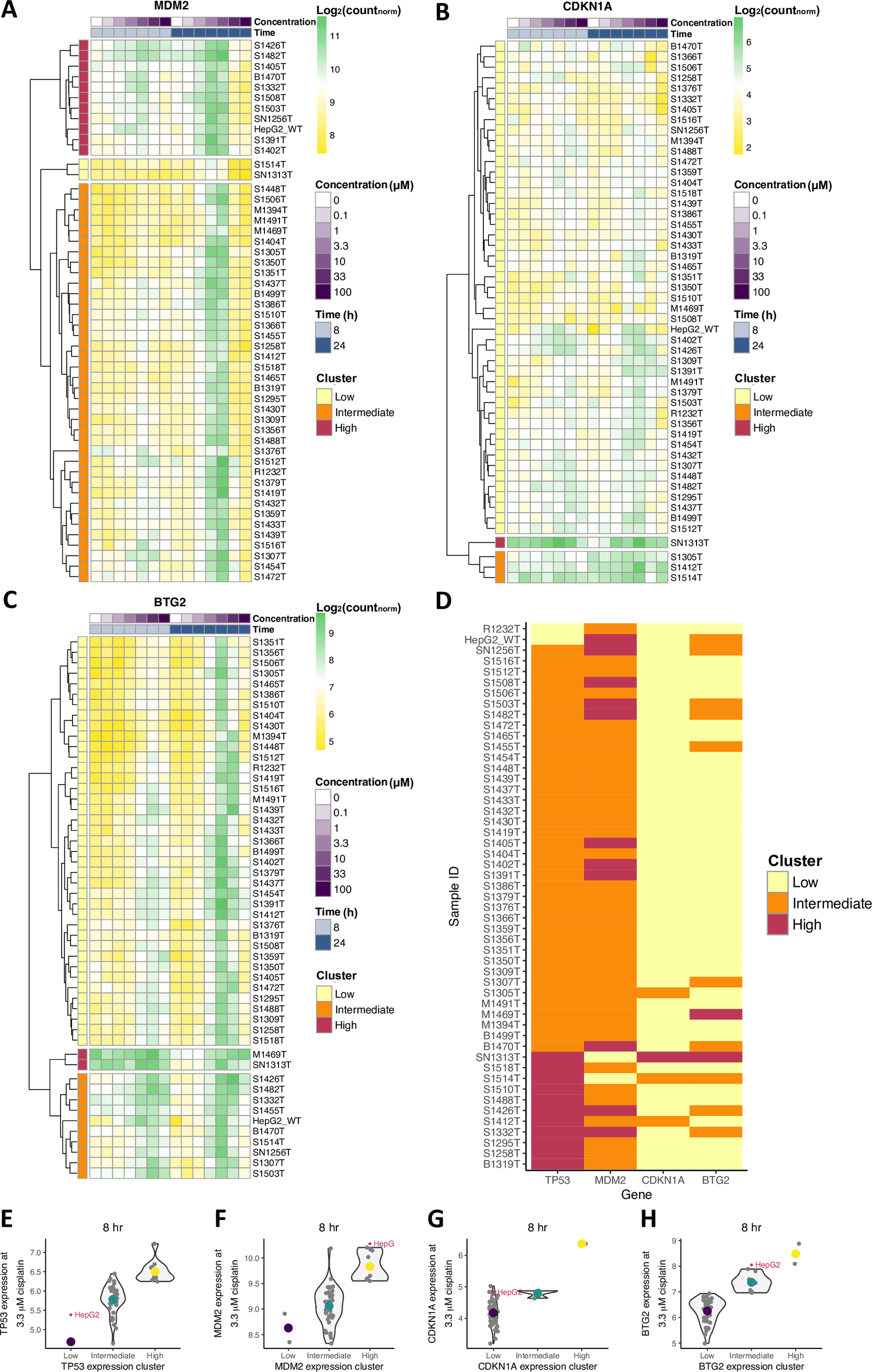

Supplement: S2 Fig — (A-C) Gene expression heatmaps for MDM2 (A), CDKN1A (B) and BTG2 (C). (D) Gene expression cluster assignment for HepG2 cells and PHH donor samples based on hierarchical clustering. Note that clusters are not conserved among genes. (E-H) Basal expression of TP53 (E), MDM2 (F), CDKN1A (G) and BTG2 (H) in PHHs and the HepG2 cell line within their corresponding low-, intermediate- and high-expression clusters. Contour lines are violin plots with individual samples marked by small grey dots, and means per cluster by large colored dots. (TIF) [file pcbi.1010264.s002.tif]

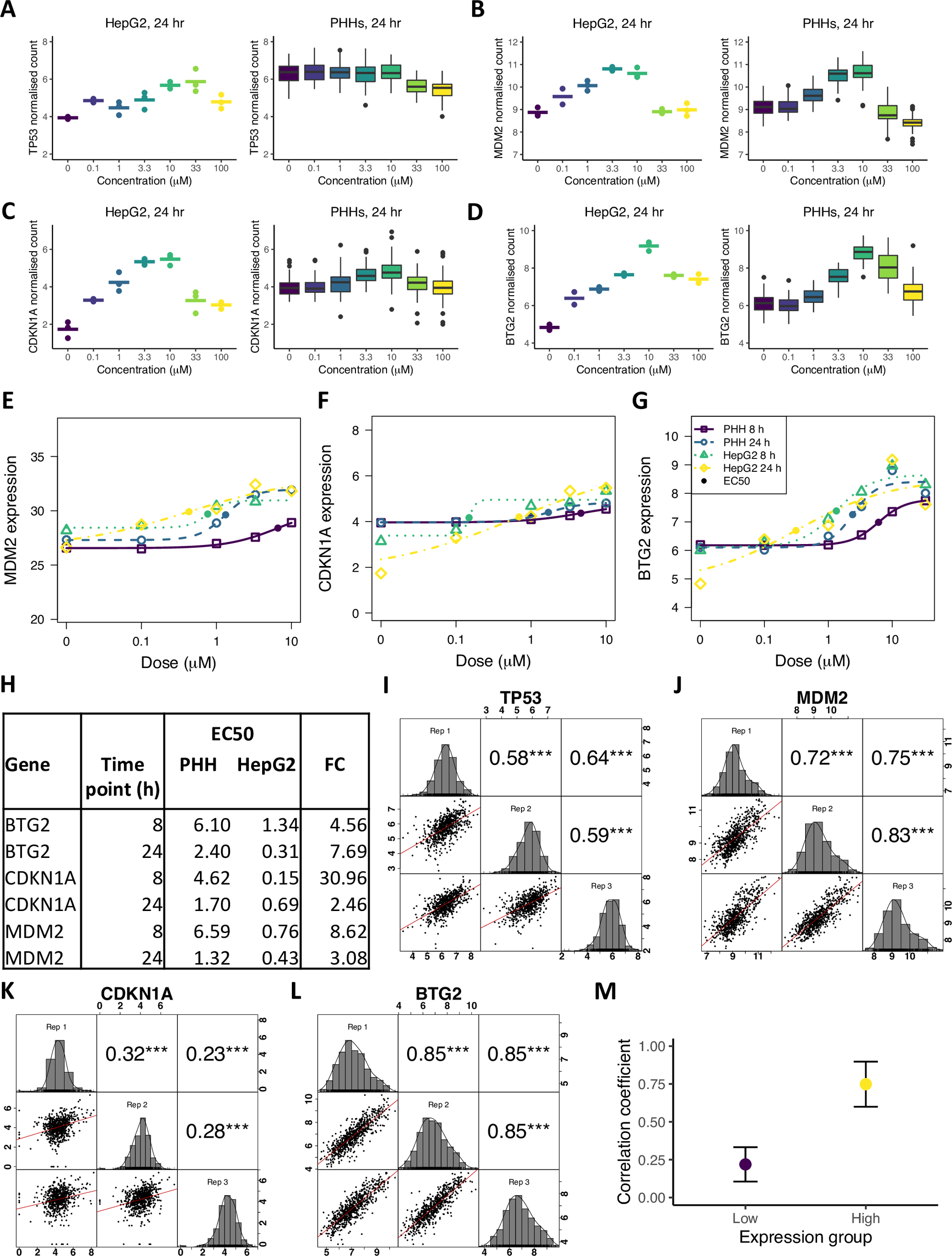

Supplement: S3 Fig — (A-D) TP53 (A), MDM2 (B), CDKN1A (C) and BTG2 (D) gene expression patterns as a function of cisplatin concentration in HepG2 cells (three replicates (dots) and their mean (line segments), left panels) and PHHs (right panels) at the 24-hour time point. Note that at high cisplatin concentrations, gene expression declines, which is likely explained by cytotoxicity onset. (E-G) Dose-response curve fits based on a Hill equation for MDM2 (E), CDKN1A (F) and BTG2 (G), with data means per condition (empty colored markers) and EC50 values (solid colored dots). (H) Fold change differences between EC50 values per gene. (I-L) Correlation plots between technical replicates of PHH measurements for TP53 (I), MDM2 (J), CDKN1A (K) and BTG2 (L). (M) Correlation coefficient between technical replicates (mean ± sd) of the 10 lowest (purple) and 10 highest (yellow) expressed genes. (TIF) [file pcbi.1010264.s003.tif]

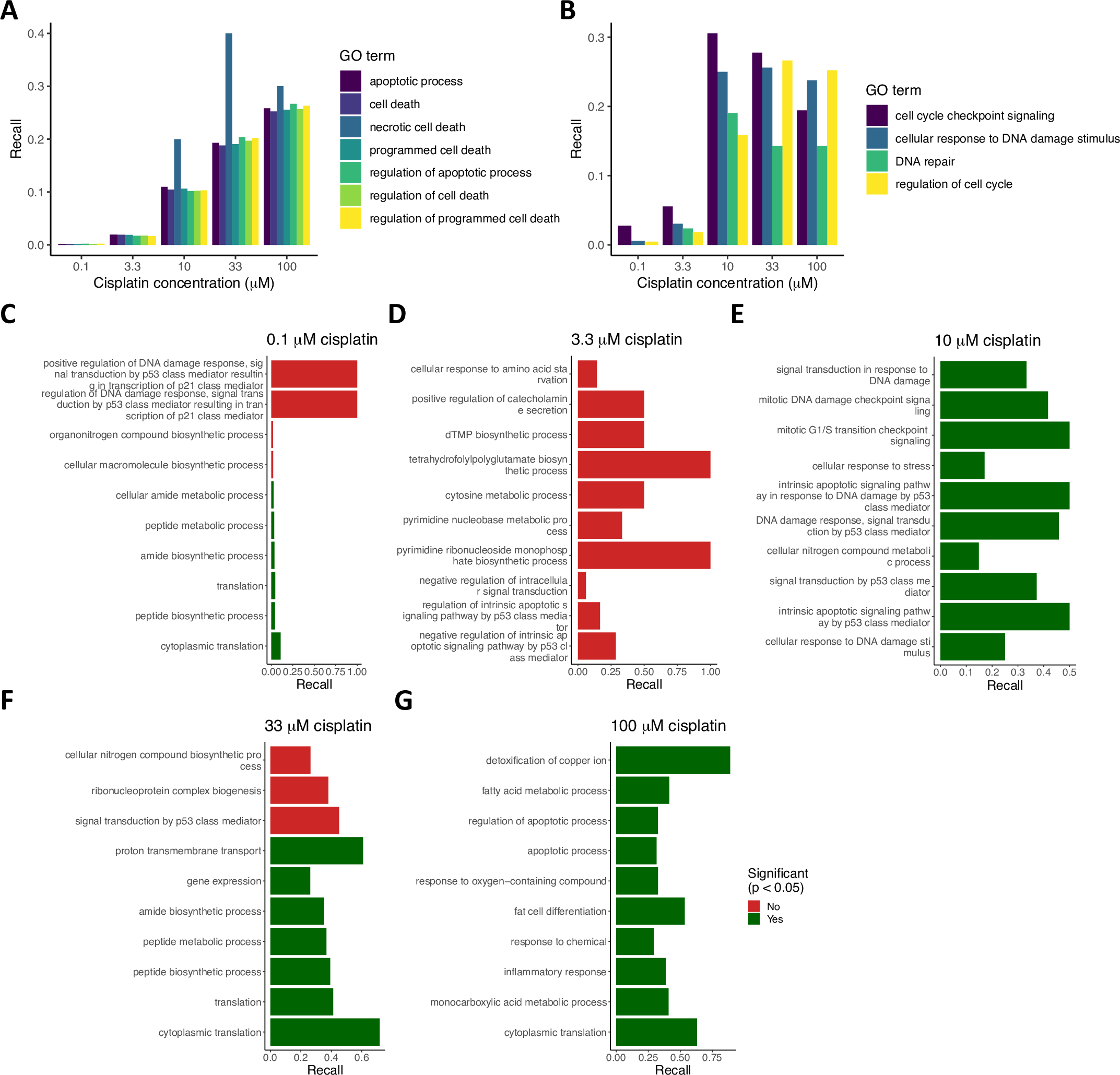

Supplement: S4 Fig — (A-B) Enrichment of selected GO terms for cell death (A) and cell health (B) terms at increasing cisplatin concentrations at the 8-hour time point. (C-G) The 10 most significant enrichment terms at 0.1 (C), 3.3 (D), 10 (E), 33 (F) and 100 (G) μM cisplatin. Note that the 1 μM condition is not included, due to the limited number of genes that passed the criteria for differentially expressed genes and the consequent absence of functional enrichment. (TIF) [file pcbi.1010264.s004.tif]

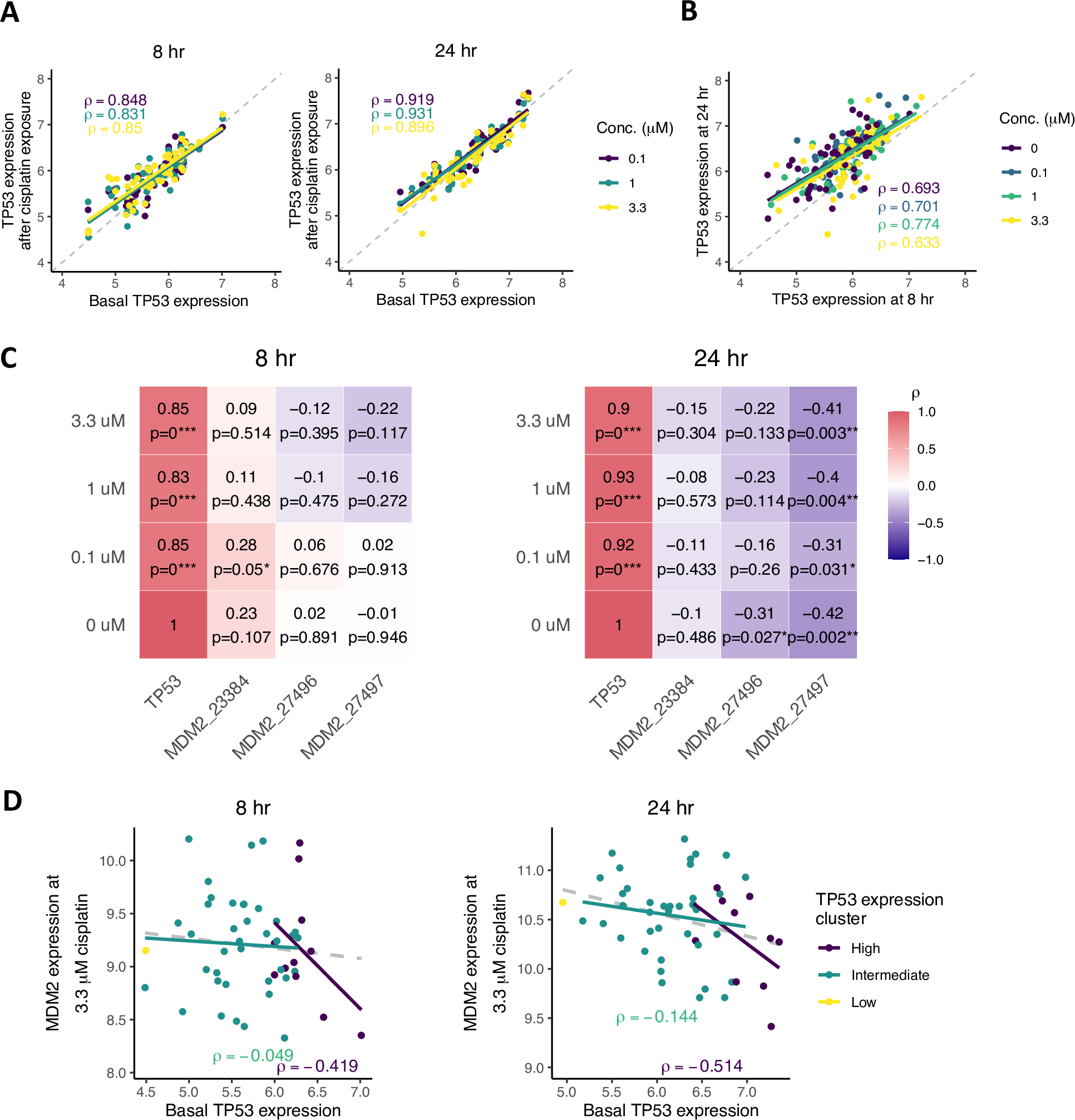

Supplement: S5 Fig — (A) Correlations of basal TP53 with TP53 expression after 0.1, 1 and 3.3 μM cisplatin exposure at 8 (left) and 24 (right) hours. (B) Correlation between TP53 expression at 8- and 24-hour timepoint. Dashed grey lines in (A-B), y = x. (C) Correlations of basal TP53 with the three probes of MDM2 (p1, p2 and p3) at 8 (left) and 24 (right) hours. (D) Correlations of basal TP53 with MDM2 (mean of the three probes) at 8 (left) and 24 (right) hours split per TP53-expression cluster. Grey dashed lines represent the overall correlation for all clusters together. (TIF) [file pcbi.1010264.s005.tif]

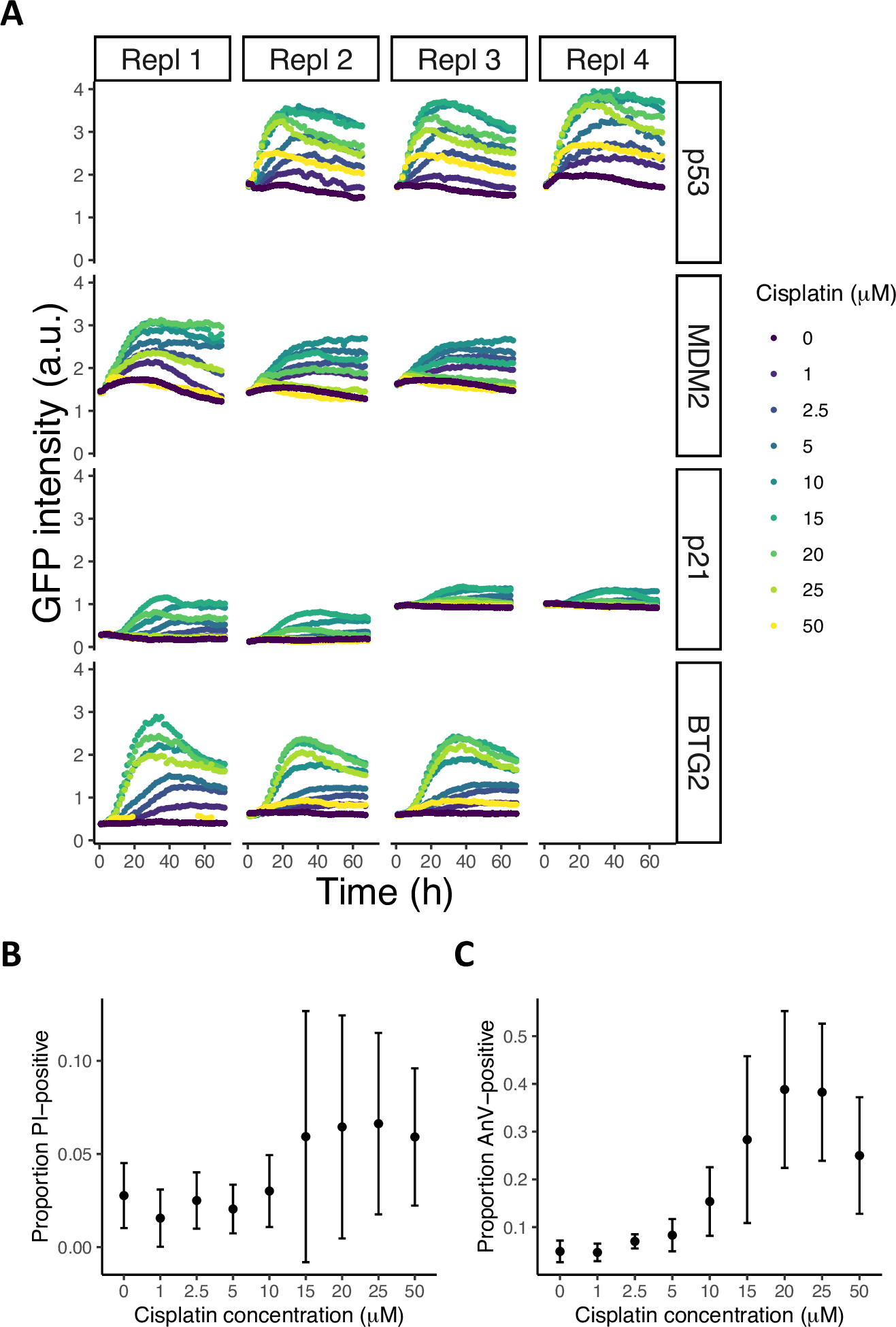

Supplement: S6 Fig — (A) Unnormalised GFP intensities for proteins and biological replicates separately. (B-C) Maximal proportion of PI-positive (B) and AnV-positive (C) cells at increasing cisplatin concentrations. Mean ± sd of all 13 experiments with the 3 to 4 replicates per BAC-GFP reporter. (TIF) [file pcbi.1010264.s006.tif]

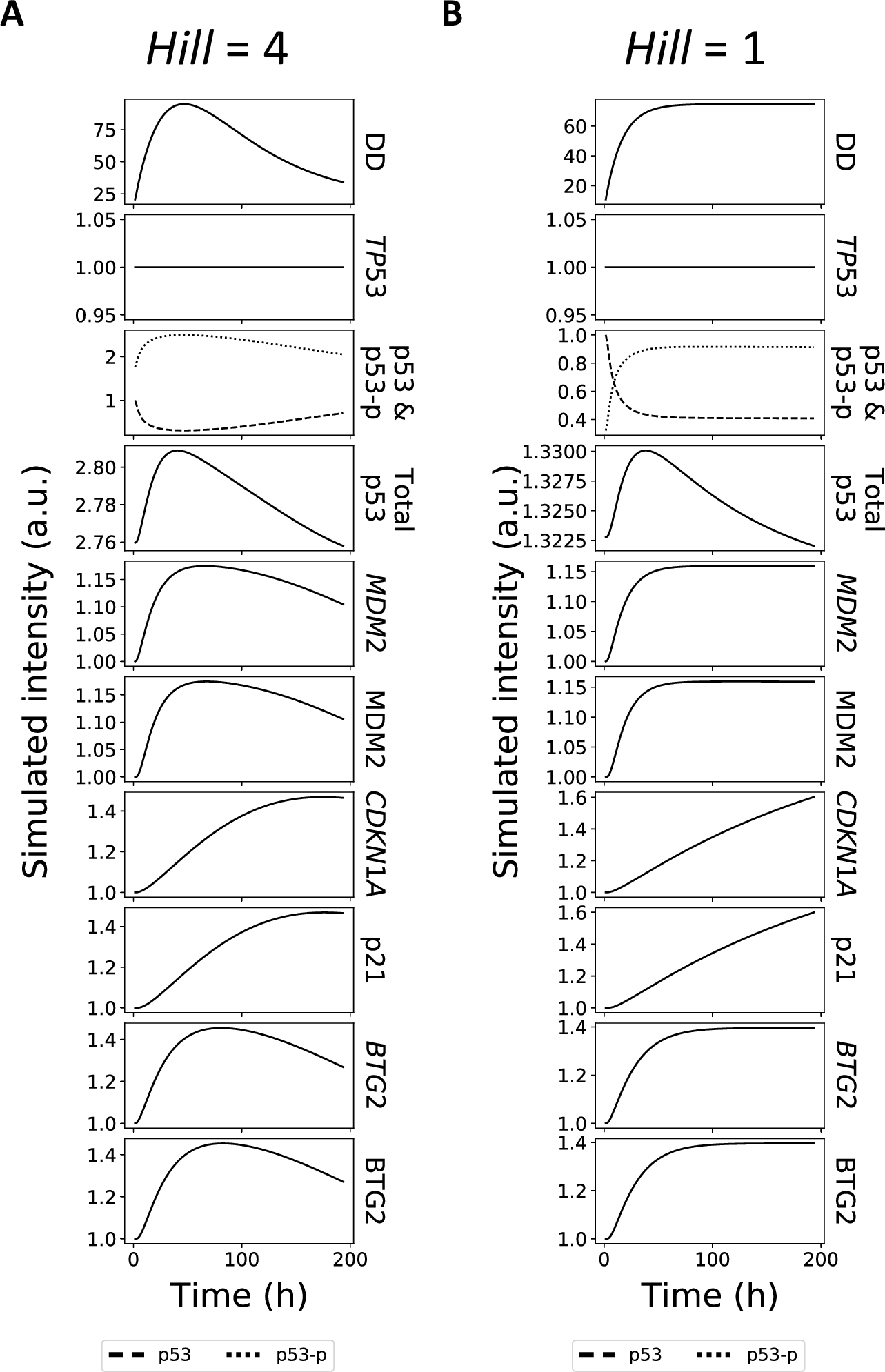

Supplement: S7 Fig — (A-B) Dynamics of the model species for a model with p53-dependent activation of transcriptional regulation with Hill parameter 4 (A) or 1 (B). DD, DNA damage. (TIF) [file pcbi.1010264.s007.tif]

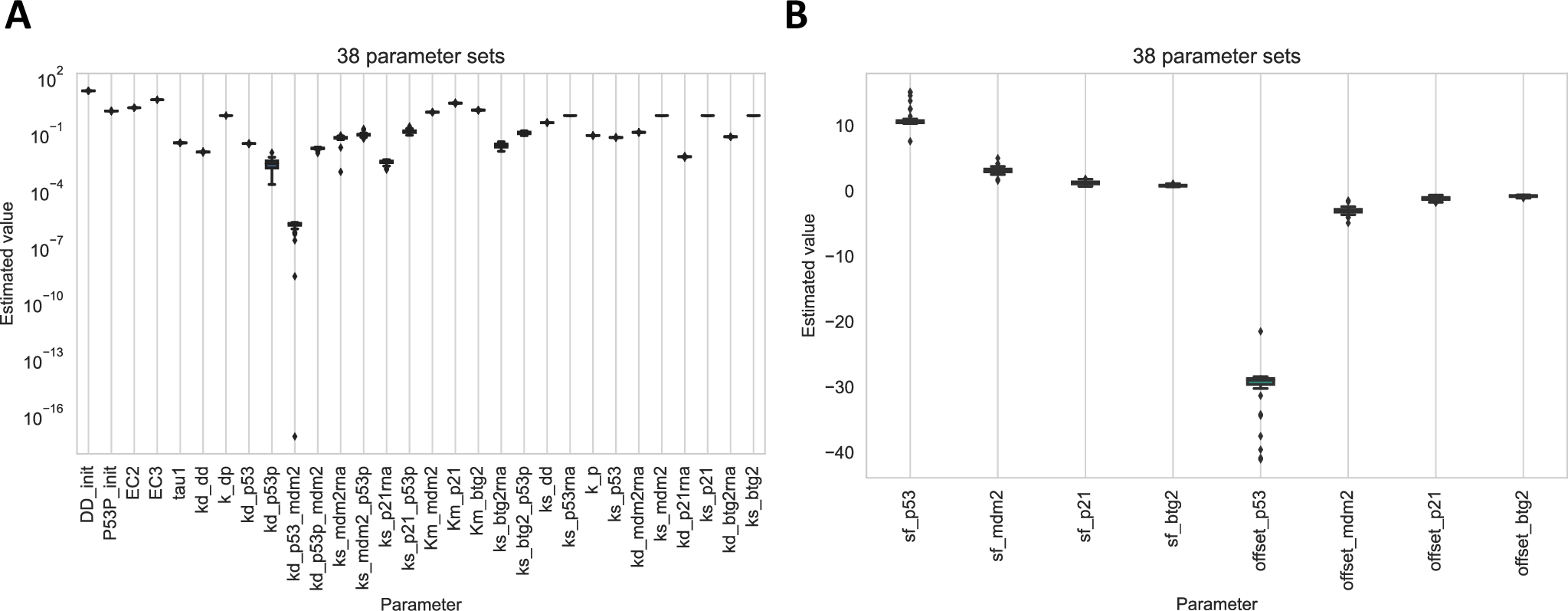

Supplement: S8 Fig — A) Estimates of the ODE model parameters and initial states on a log10 scale. B) Estimates of the scaling and offset parameters used in the observable function on a linear scale. (TIF) [file pcbi.1010264.s008.tif]

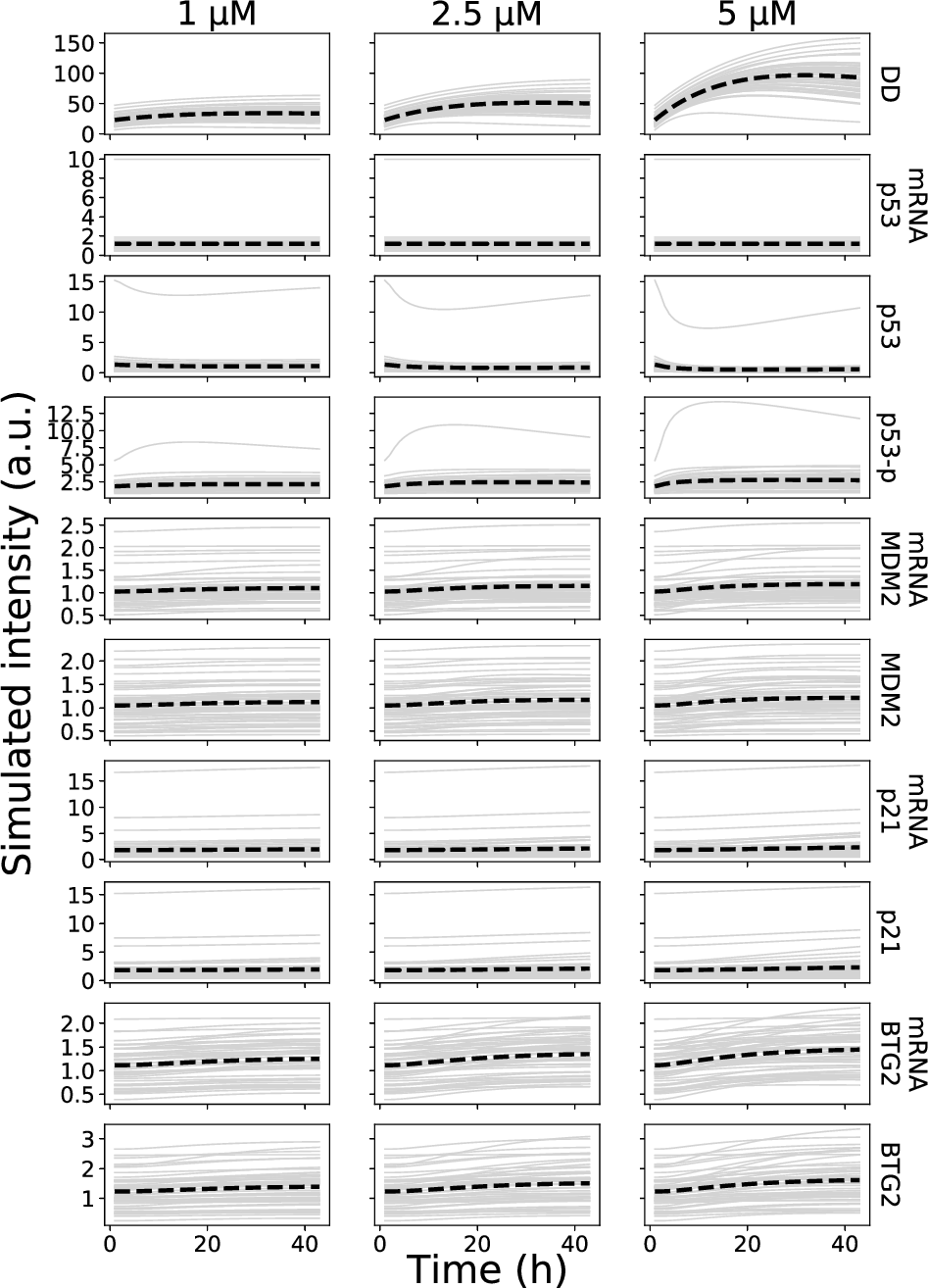

Supplement: S9 Fig — (TIF) [file pcbi.1010264.s009.tif]

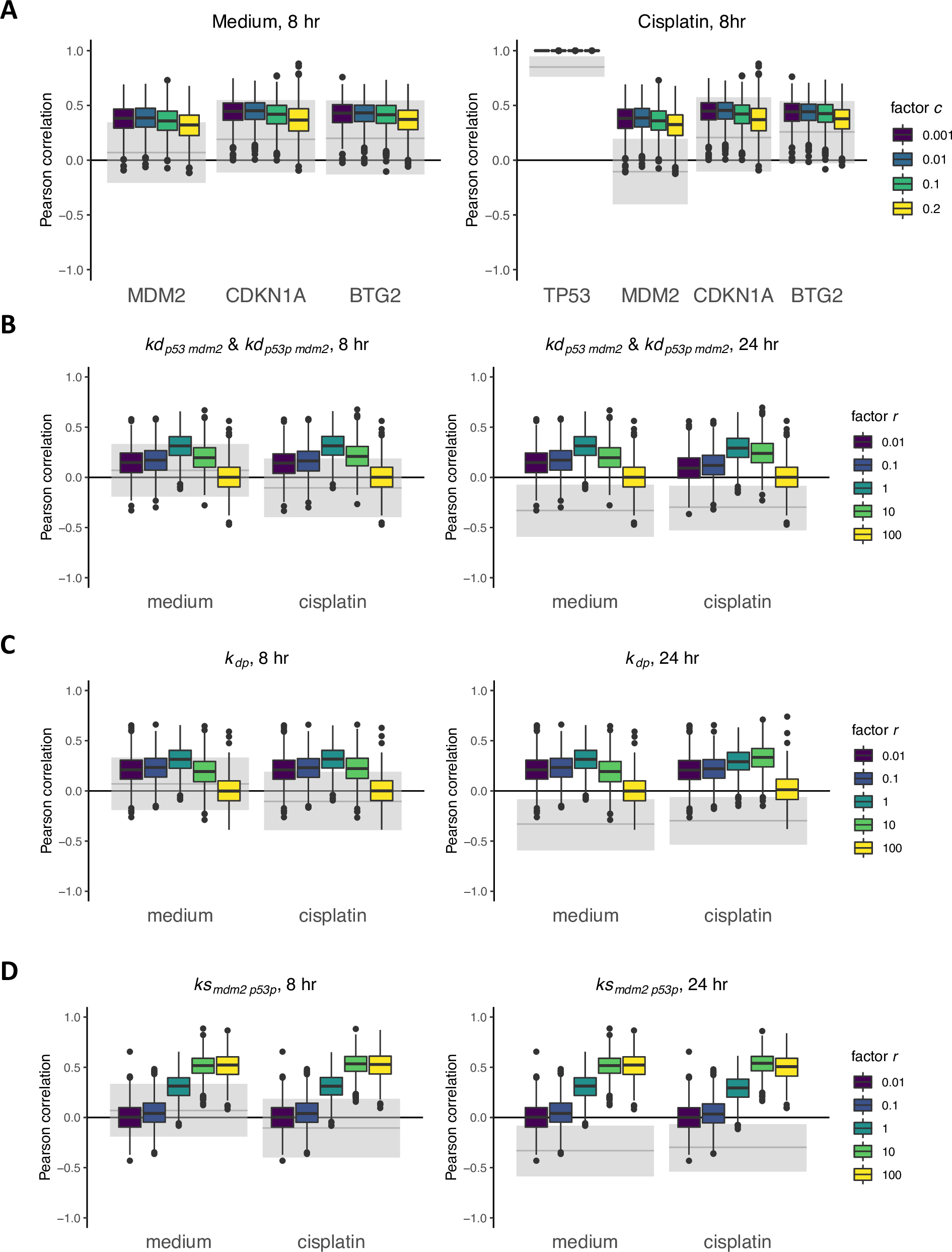

Supplement: S10 Fig — (A) Correlations between basal TP53 expression and the downstream targets MDM2, CDKN1A and BTG2 at 8 hours for HepG2-derived virtual samples (colored boxes) and PHHs (grey shaded areas) in basal expression conditions (left) and after 3.3 μM cisplatin exposure (right). Increasing variability in parameter values, i.e., increasing factor c, improves the match between the correlations in the HepG2-derived virtual samples and the PHH donor samples. (B-D) Effect of changes in MDM2 feedback strength (B), dephosphorylation rate (C) and p53-dependent MDM2 synthesis rate (D) on correlation between TP53 and MDM2 after 8 (left) and 24 (right) hours. Correlations are shown for basal TP53 expression with MDM2 expression in medium or after 3.3 μM cisplatin exposure at varying parameter multiplication factors r. A value r = 1 implies no adjustment of the parameter value with respect to the fitted values, r > 1 implies stronger feedback and r < 1 implies weaker feedback. Colored boxes are the correlations found for HepG2-derived virtual samples. Grey horizontal lines represent the measured correlation for the 50 PHH donor samples. Grey shaded areas represent the 95% confidence interval of the correlation measurements acquired with 1000 times bootstrapping. (TIF) [file pcbi.1010264.s010.tif]

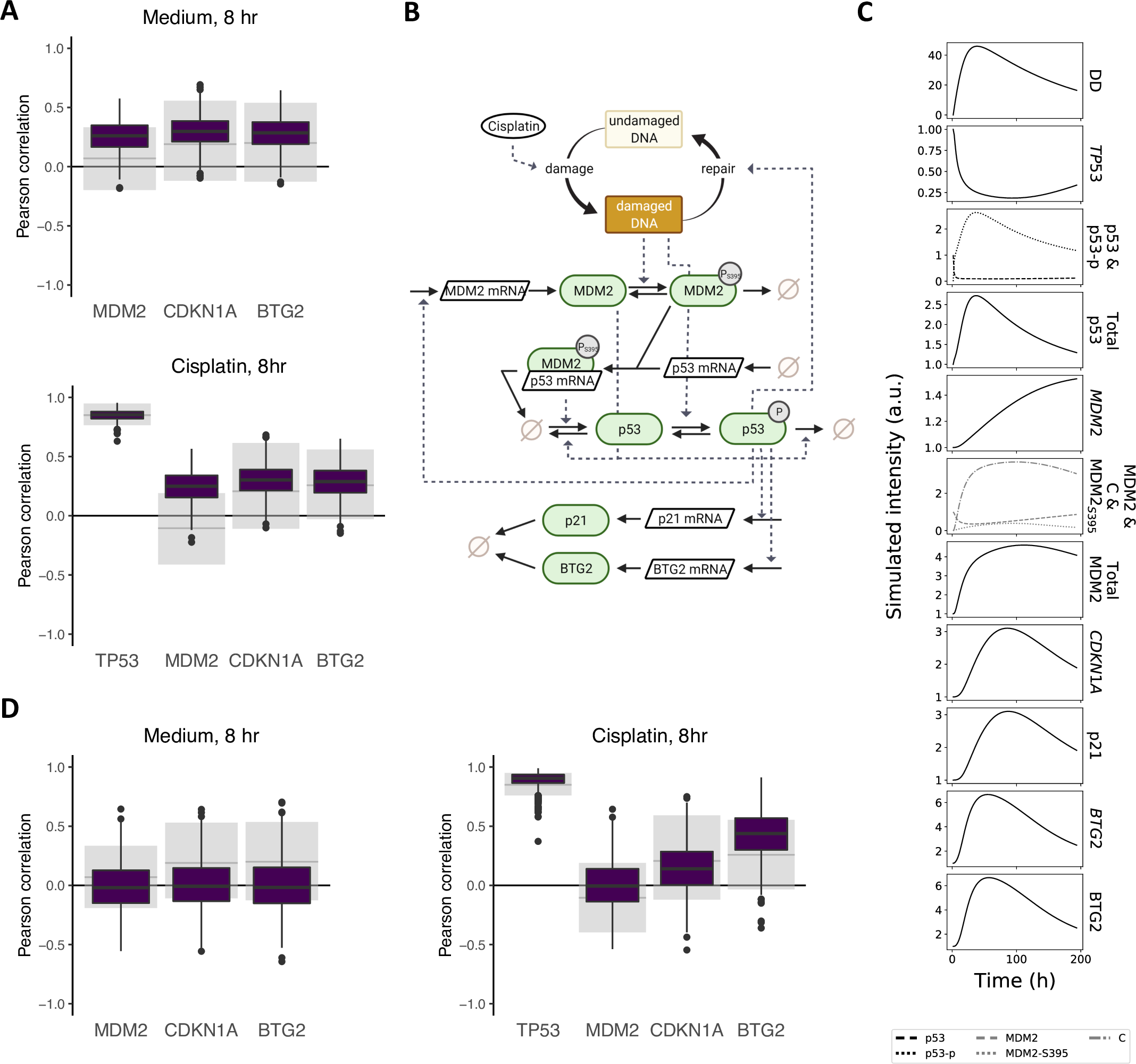

Supplement: S11 Fig — (A) Correlations between basal TP53 expression and the downstream targets MDM2, CDKN1A and BTG2 at 8 hours for HepG2-derived virtual samples (colored boxes), created with a model with a non-linear p53-MDM2 feedback. (B) Alternative model in which p53 mRNA binds to phosphorylated MDM2 and induces expression of p53 mRNA. (C) Simulation of the alternative model (as in B) after parameter estimation. (D) Correlations between basal TP53 expression and the downstream targets MDM2, CDKN1A and BTG2 at 8 hours for HepG2-derived virtual samples (colored boxes), created with the alternative model (as in B). Correlations in B and D are shown for basal expression conditions (left) and after 3.3 μM cisplatin exposure (right) with variability factor c = 0.1 and a small amount of measurement noise for B, and variability factor c = 0.2 without measurement noise for D. Moreover, in B and D the grey solid lines represent the observed correlations in PHHs and the grey shaded areas represent the 95% confidence interval of correlations found for alternative PHH donor sets acquired with 1000 times bootstrapping. (TIF) [file pcbi.1010264.s011.tif]
