# Supplementary material for: Model-based translation of DNA damage signaling dynamics across cell types
Source: PLoS Comput Biol. 2022 Jul 8;18(7):e1010264. doi: 10.1371/journal.pcbi.1010264 (PMC9269748; doi:10.1371/journal.pcbi.1010264)
Supplement: S2 Table — Fixed parameter values are indicated with a diamond (⋄) and parameters that are determined with steady state constraint calculations with a star (⋆). (DOCX) [file pcbi.1010264.s013.docx]

**S2 Table**. Parameter description and estimated values for the alternative DDR model described in S1 Methods. Fixed parameter values are indicated with a diamond (⋄) and parameters that are determined with steady state constraint calculations with a star (⋆).

| **Parameter** | **Unit** | **Description** | **Estimated values** |
| --- | --- | --- | --- |
| DD_init_ | - | initial amount of DNA damage | 0 ⋄ |
| P53 RNA_init_ | au | initial amount of p53 mRNA (*TP53*) | 1 ⋄ |
| P53_init_ | au | initial amount of p53 | 1 ⋄ |
| P53P_init_ | au | initial amount of p53-p | 0 ⋄ |
| MDM2 RNA_init_ | au | initial amount of MDM2 mRNA (*MDM2*) | 1 ⋄ |
| P21 RNA_init_ | au | initial amount of p21 mRNA (*CDKN1A*) | 1 ⋄ |
| BTG2 RNA_init_ | au | initial amount of BTG2 mRNA (*BTG2*) | 1 ⋄ |
| MDM2_init_ | au | initial amount of MDM2 | 1 ⋄ |
| MDM2S395_init_ | au | initial amount of MDM2-S395 | 0 ⋄ |
| C_nit_ | au | initial amount of complex C | 0 ⋄ |
| P21_init_ | au | initial amount of p21 | 1 ⋄ |
| BTG2_init_ | au | initial amount of BTG2 | 1 ⋄ |
| EC_1_ | au · hr^-1^ | effective concentration for 1 $\mu$M applied concentration | 0.667 ⋄ |
| EC_2_ | au · hr^-1^ | effective concentration for 2.5 $\mu$M applied concentration | 1.525 |
| EC_3_ | au · hr^-1^ | effective concentration for 5 $\mu$M applied concentration | 3.069 |
| $\tau$ | hr^-1^ | cisplatin decay rate | 0.016 |
| kd­_p53 RNA_ | hr^-1^ | degradation rate of p53 mRNA (*TP53*) | 0.667 ⋄ |
| kd_mdm2_ | hr^-1^ | degradation rate of MDM2 | 0.667 ⋄ |
| kd_p21_ | hr^-1^ | degradation rate of p21 | 0.667 ⋄ |
| kd_btg2_ | hr^-1^ | degradation rate of BTG2 | 0.667 ⋄ |
| kd­_p53_ | hr^-1^ | degradation rate of p53 | 0.667 ⋄ |
| kd_dd_ | au^-1^ · hr^-1^ | DNA damage repair rate | 0.014 |
| k_dp_ | hr^-1^ | dephosphorylation rate of p53-p | 28.253 |
| kd_p53p_ | hr^-1^ | degradation rate of p53-p | 0.135 |
| kd_p53 mdm2_ | au^-1^ · hr^-1^ | MDM2-dependent degradation rate of p53 | 2.027 |
| kd_p53p mdm2_ | au^-1^ · hr^-1^ | MDM2-dependent degradation rate of p53-p | 2.177 |
| ks_mdm2 p53p_ | au · hr^-1^ | maximal p53-p-dependent synthesis rate of MDM2 | 0.005 |
| Km_mdm2_ | au | Michaelis-Menten constant for MDM2 | 1.533 |
| ks_p53 C_ | hr^-1^ | complex-dependent synthesis of p53 | 0.574 |
| kb | au^-1^ · hr^-1^ | binding constant of MDM2-S395 to p53 mRNA | 7.494 |
| ka | hr^-1^ | phosphorylation rate of MDM2 | 0.031 |
| kd_C_ | hr^-1^ | degradation rate of complex C | 0.148 |
| k_p_ | hr^-1^ | phosphorylation rate of p53 | 17.854 |
| kd_mdm2 RNA_ | hr^-1^ | degradation rate of MDM2 mRNA (*MDM2*) | 0.001 |
| ks_p21 RNA_ | au · hr^-1^ | basal synthesis rate of p21 mRNA (*CDKN1A*) | 0.020 |
| ks_p21 p53p_ | au · hr^-1^ | maximal p53-p-dependent synthesis rate of p21 | 0.098 |
| Km_p21_ | au | Michaelis-Menten constant for p21 | 2.213 |
| Km_btg2_ | au | Michaelis-Menten constant for BTG2 | 1.711 |
| ks_btg2 RNA_ | au · hr^-1^ | basal synthesis rate of BTG2 mRNA (*BTG2*) | 0.091 |
| ks_btg2 p53p_ | au · hr^-1^ | maximal p53-p-dependent synthesis rate of BTG2 | 0.632 |
| ks_p53 RNA_ | au · hr^-1^ | synthesis rate of p53 mRNA (*TP53*) | 0.667 ⋆ |
| ks_p53_ | hr^-1^ | synthesis rate of p53 | 2.694 ⋆ |
| ks_mdm2 RNA_ | au · hr^-1^ | basal synthesis rate of MDM2 mRNA (*MDM2*) | 0.001 ⋆ |
| ks_mdm2_ | hr^-1^ | synthesis rate of MDM2 | 0.667 ⋆ |
| kd_p21 RNA_ | hr^-1^ | degradation rate of p21 mRNA (*CDKN1A*) | 0.020 ⋆ |
| ks_p21_ | hr^-1^ | synthesis rate of p21 | 0.667 ⋆ |
| kd_btg2 RNA_ | hr^-1^ | degradation rate of BTG2 mRNA (*BTG2*) | 0.091 ⋆ |
| ks_btg2_ | hr^-1^ | synthesis rate of BTG2 | 0.667 ⋆ |
| scaling_p53_ | - | scaling factor for total p53 | 0.306 |
| scaling_mdm2_ | - | scaling factor for MDM2 | 0.176 |
| scaling_p21_ | - | scaling factor for p21 | 0.149 |
| scaling_btg2_ | - | scaling factor for BTG2 | 0.065 |
| offset _p53_ | au | offset for total p53 | -0.270 |
| offset _mdm2_ | au | offset for MDM2 | -0.093 |
| offset _p21_ | au | offset for p21 | -0.087 |
| offset _btg2_ | au | offset for BTG2 | -0.040 |
