# Supplementary material for: Model-based translation of DNA damage signaling dynamics across cell types
Source: PLoS Comput Biol. 2022 Jul 8;18(7):e1010264. doi: 10.1371/journal.pcbi.1010264 (PMC9269748; doi:10.1371/journal.pcbi.1010264)
Supplement: S1 Methods — (DOCX) [file pcbi.1010264.s014.docx]

**Model with alternative non-linear feedback of MDM2 on p53**

For the model with non-linear feedback from MDM2 on p53, we changed the ODEs that describe the dynamics of p53 and p53-p (Equations 4-5 in the main text) as follows:

|  | $\frac{dP53}{\mathrm{dt}} =\mathrm{ks}_{p53}\cdot{P53}_{\mathrm{RNA}}+k_{\mathrm{dp}}\cdot{P53}_{p}-{k_{p}\cdot P53\cdot DD-kd}_{p53}\cdot P53-\mathrm{kd}_{p53 mdm2}\cdot\frac{{P53}^{4}\cdot{MDM2}^{4}}{1+ {P53}^{4}+ {MDM2}^{4}}$ | S.Eq. 1 |
| --- | --- | --- |

|  | $\frac{d{{P53}_{p}}}{\mathrm{dt}} ={k_{p} \cdot P53 \cdot DD - k_{\mathrm{dp}} \cdot{P53}_{p} - kd}_{p53p} \cdot{P53}_{p}-\mathrm{kd}_{p53p mdm2}\cdot\frac{{{P53}_{p}}^{4}\cdot{MDM2}^{4}}{1+ {{P53}_{p}}^{4}+ {MDM2}^{4}}$ | S.Eq. 2 |
| --- | --- | --- |

**Alternative model for p53 signaling**

We designed an alternative model for the p53 signaling pathway which incorporated a positive feedback from MDM2 on p53 in addition to the negative feedback. Here, MDM2 is phosphorylated at residue Serine 395 (*MDM2_S395_*) and subsequently binds to p53 mRNA to form a complex *C* which promotes translation of p53 [1,2]. Furthermore, we used inner model species for which we had no experimental data to constrain the dynamics. Therefore, we assumed that there was no constitutive DNA damage in steady state, i.e., we set $\mathrm{ks}_{\mathrm{DD}}$ to 0:

|  | $\frac{\mathrm{dDD}}{\mathrm{dt}} =-\mathrm{kd}_{\mathrm{DD}} \cdot DD \cdot{P53}_{p}+S$. | S.Eq. 3 |
| --- | --- | --- |

We described *P53_RNA_* and *P53* as in our default DDR model, but in addition included the binding of *P53_RNA_* to *MDM2_S395_* into complex *C* in the equation of *P53_RNA_* with rate parameter *kb* (S.Eq. 4). The formation of p53 protein was now also dependent on *C*, with parameter *ks_p53 C_* (S.Eq. 5). We kept the ODEs for *P53P* and *MDM2_RNA_* the same (S.Eq. 6-7), but added DNA damage dependent phosphorylation of MDM2 at rate $ka$ to its ODE (S.Eq. 8). We added supplementary equations 9 and 10 to describe the dynamics of *MDM2_S395_* and complex *C*, where *kd_C_* is the degradation rate of the complex. Equations for p21 and BTG2 mRNA and protein species were the same as before. The ODEs of the alternative model thus became:

|  | $\frac{d{P53}_{\mathrm{RNA}}}{\mathrm{dt}} =\mathrm{ks}_{p53 RNA} -\mathrm{kd}_{p53 RNA} \cdot{P53}_{\mathrm{RNA}}-kb\cdot{MDM2}_{S395}\cdot{P53}_{\mathrm{RNA}}$, | S.Eq. 4 |
| --- | --- | --- |

|  | $\frac{dP53}{\mathrm{dt}} =\mathrm{ks}_{p53}\cdot{P53}_{\mathrm{RNA}}+\mathrm{ks}_{p53 c}\cdot C+k_{\mathrm{dp}}\cdot{P53}_{p}-{k_{p}\cdot P53\cdot DD-kd}_{p53}\cdot P53-\mathrm{kd}_{p53 mdm2}\cdot P53\cdot MDM2$, | S.Eq. 5 |
| --- | --- | --- |

|  | $\frac{d{{P53}_{p}}}{\mathrm{dt}} ={k_{p} \cdot P53 \cdot DD - k_{\mathrm{dp}} \cdot{P53}_{p} - kd}_{p53p} \cdot{P53}_{p}-\mathrm{kd}_{p53p mdm2}\cdot{P53}_{p}\cdot MDM2$, | S.Eq. 6 |
| --- | --- | --- |

|  | $\frac{{dMDM2}_{\mathrm{RNA}}}{\mathrm{dt}}= \mathrm{ks}_{mdm2 RNA}+\frac{\mathrm{ks}_{mdm2 p53p}\cdot{{P53}_{p}}^{4}}{\mathrm{Km}_{mdm2}^{4}+{{P53}_{p}}^{4}}-\mathrm{kd}_{mdm2 RNA}\cdot{MDM2}_{\mathrm{RNA}}$, | S.Eq. 7 |
| --- | --- | --- |

|  | $\frac{dMDM2}{\mathrm{dt}}= \mathrm{ks}_{mdm2}\cdot{MDM2}_{\mathrm{RNA}}-\mathrm{kd}_{mdm2}\cdot MDM2-\mathrm{ka}\cdot MDM2\cdot\mathrm{DD}$, | S.Eq. 8 |
| --- | --- | --- |

|  | $\frac{d{MDM2}_{S395}}{\mathrm{dt}}= \mathrm{ka}\cdot MDM2\cdot DD- kb\cdot{MDM2}_{S395}\cdot{P53}_{\mathrm{RNA}}$, | S.Eq. 9 |
| --- | --- | --- |

|  | $\frac{\mathrm{dC}}{\mathrm{dt}}= kb\cdot{MDM2}_{S395}\cdot{P53}_{\mathrm{RNA}} -\mathrm{kd}_{C}\cdot C$, | S.Eq. 10 |
| --- | --- | --- |

|  | $\frac{{dP21}_{\mathrm{RNA}}}{\mathrm{dt}} = \mathrm{ks}_{p21 RNA}+\frac{\mathrm{ks}_{p21 p53p}\cdot{{P53}_{p}}^{4}}{\mathrm{Km}_{p21}^{4}+{{P53}_{p}}^{4}}-\mathrm{kd}_{p21 RNA}\cdot{P21}_{\mathrm{RNA}}$, | S.Eq. 11 |
| --- | --- | --- |

|  | $\frac{dP21}{\mathrm{dt}} = \mathrm{ks}_{p21}\cdot{P21}_{\mathrm{RNA}}-\mathrm{kd}_{p21}\cdot P21$, | S.Eq. 12 |
| --- | --- | --- |

|  | $\frac{{dBTG2}_{\mathrm{RNA}}}{\mathrm{dt}} = \mathrm{ks}_{btg2 RNA}+\frac{\mathrm{ks}_{btg2 p53p}\cdot{{P53}_{p}}^{4}}{\mathrm{Km}_{btg2}^{4}+{{P53}_{p}}^{4}}-\mathrm{kd}_{btg2 RNA}\cdot{BTG2}_{\mathrm{RNA}}$, and | S.Eq. 13 |
| --- | --- | --- |

|  | $\frac{dBTG2}{\mathrm{dt}} = \mathrm{ks}_{btg2}\cdot{BTG2}_{\mathrm{RNA}}-\mathrm{kd}_{btg2}\cdot BTG2$. | S.Eq. 14 |
| --- | --- | --- |

The parameter values for this alternative DDR model can be found in S2 Table.

**References**

1. Gajjar M, Candeias MM, Malbert-Colas L, Mazars A, Fujita J, Olivares-Illana V, et al. The p53 mRNA-Mdm2 interaction controls Mdm2 nuclear trafficking and is required for p53 activation following DNA damage. Cancer Cell. 2012;21: 25–35.

2. Candeias MM, Malbert-Colas L, Powell DJ, Daskalogianni C, Maslon MM, Naski N, et al. P53 mRNA controls p53 activity by managing Mdm2 functions. Nat Cell Biol. 2008;10: 1098–1105.
