# Supplementary material for: Model-based translation of DNA damage signaling dynamics across cell types
Source: PLoS Comput Biol. 2022 Jul 8;18(7):e1010264. doi: 10.1371/journal.pcbi.1010264 (PMC9269748; doi:10.1371/journal.pcbi.1010264)
Supplement: Attachment — Submitted filename: Heldring_R1_responsetoreviewers.pdf [file pcbi.1010264.s015.pdf]

Reviewer #1

The authors create a mathematical model to predict dynamics of p53 mRNA and protein levels as well as protein levels of select p53 target genes in response to cisplatin induced DNA damage. Based on experiments conducted in HepG2 cells and primary human hepatocytes (PHH) the authors created a set of virtual PHHs to test this model. Thereby, the moderate correlations that the authors found in 50 biological PHH donor samples for p53, p21, and BTG2, was resembled in the virtual samples. The negative correlation between p53 and Mdm2 in the PHH donor samples could not be replicated with the model however. The authors conclude that HepG2-based computational modelling can be accurate for some, but not all DDR elements.
To frame our comments, we point out that we are molecular biologists, focussing on in vitro and in vivo investigations of the p53-Mdm2 feedback mechanism rather than computational/mathematical biologists. Therefore, we comment mainly on the biological context of the experiments and their findings. In general, the paper is well written and at each necessary point, the authors commit to its shortcomings and limitations, including its biological context. As such, the manuscript is well reflected and discussed with the context of currently available p53-Mdm2 literature (in HepG2 cells). We are aware that computational modelling of real-world problems is a challenging task and it comes along with simplifications. And we also believe that meeting these challenges is a valuable starting point for future developments.
Still, from the molecular biological point of view we want to raise the following concerns in response to the manuscript:

We thank the reviewer for the complements regarding our clear and fair presentation of our work, and for bringing in their experimentalist point of view.

Major concerns

1. The authors do not provide direct evidence for DNA damage. Given the large disparity in gene expression in the response to cisplatin treatment in HepG2 and PHH cells, raises the question if DNA damage is inducible to equal extent in both systems. Comparing immortalized cell lines with timely limited culturable primary cells could lead to DNA damage-independent, but still p53-mediated, cellular responses. Therefore, we suggest showing direct evidence for DNA damage in both systems with the same concentrations as used in the manuscript i.e., gH2Ax induction, comet assay, 8OH-immunofluorescence or if available to provide a reference showing cisplatin-induced DNA damage in HepG2 cells or direct experimental evidence.

The reviewer is correct that we did not provide proof for equal extent of DNA damage in HepG2 cells and PHHs. Cisplatin is known to cause inter- and intrastrand DNA adducts that lead to secondary single stranded and double stranded breaks. Indeed, the extent, timing and repair rate of these different DNA damage types can differ between cell types, which should be considered when experimentally comparing the amount of damage. In our study, we investigated whether the pathway dynamics in HepG2 cells are likely comparable to dynamics in PHHs at equal exposure levels to cisplatin, even though the sensitivity to cisplatin may differ between the cell types. Nevertheless, it is known that cisplatin inflicts DNA damage to both HepG2 and PHH, although different assays are not equally sensitive for damage in the different cell types (http://dx.doi.org/10.1007/s00204-020-02736-z,

http://dx.doi.org/10.1080/15287394.2020.1822972). In our revised manuscript, we confirm the presence of DNA damage in HepG2 cells by quantifying γH2AX induction in this cell line (shown in Fig S1A). In addition, we overlayed the cisplatin-induced gene expression of the entire S1500+ gene set for both HepG2 cells and PHHs with the whole-genome PHH gene expression in the TXG-MAPr. This highlighted an overlap in TP53 and DNA damage associated modules (Fig S1E), of which some also have a high eigengene score in the PHH TXG-MAPr for etoposide, i.e., another DNA-damaging compound. We also added discussion on this issue in our revised manuscript.

2. Although addressed as shortcoming in the text, we are concerned for the predictive capacity for CDKN1A, as the technical variability (Fig S3E-H) in the TempO-Seq analysis is quite high. How do the authors argue to utilize the data e.g., for CDKN1A with a Pearson correlation between 0.23 and 0.32, for a predictive model?

Even though the correlation between the technical replicates for CDKN1A is low, we think that it is unlikely that this is due to experimental error. Rather, this low correlation seems due to the low overall expression of CDKN1A compared to that of TP53, MDM2 and BTG2. Thus, we think it is justified to use the mean expression value, which generates a more reliable estimate for gene expression. To confirm our hypothesis that the measurement error for lowly expressed genes is relatively high, thus leading to low correlations between technical replicates, we now show the correlation plots for technical replicates of the ten lowest and highest expressed genes in the dataset and added a figure to illustrate this as supplementary material (Fig. S3M).

3. The conclusion of chapter one needs rework given the graphs shown in Figure 2. An objective measure (statistical value) for describing the data in Figure 2 (expression patterns of TP53, MDM2, CDKN1A and BTG2 in HepG2 compared to PHHs) should be provided, rather than merely stating that patterns are "similar".

We agree with the reviewer that our statement lacked an objective measure. Therefore, we have fitted dose-response curves for every gene to be able to compare the EC50 for these genes between HepG2 and PHHs and modified Figure 2 accordingly. We have included additional text in the Methods and Results section to clarify our approach.

4. The experimental conditions change from 8h and 24h to 48h and beyond. Still the derived model predicts dynamics starting at timepoint zero, which was only experimentally obtained for mRNA data. We are inconclusive if such fundamentally different biological entities (mRNA, protein) experimentally measured at different timepoints should be integrated into one model. The authors are aware of pulsatile p53 mRNA regulation (or mRNA regulation in general as referenced in the text) and concluded in the first chapters, that PHH p53 mRNA levels were non-predictive. We ask to clarify how the TempO-Seq data is reflected in the equations.

We think this question is related to a misunderstanding regarding the data on which our model is calibrated. We did this only based on time-resolved protein expression data, because the transcriptomics data contain too few time points (8h and 24h post cisplatin exposure) to constrain a model. Imaging for quantification of protein expression started

within one hour after exposure to cisplatin and continued for 65 - 72 hours, and thus our measurements contain sufficient data for early and late timepoints. Thus, we use our model that describes protein dynamics in HepG2 to generate expected dynamics for sets of virtual PHH samples, and compare these to the available mRNA measurements at 8h and 24h timepoint in real PHHs. The reviewer rightfully notices that mRNA expression in our model is only included for p53, but not MDM2, p21 and BTG2, which was likely the main factor that confused the reviewer. To address this along with the comment in point 6 about the missing mRNA predictions in our model, we modified the model to include mRNA species for MDM2, p21 and BTG2. We adjusted the Methods and Results sections accordingly and think that this takes away the unclarity with respect to the data included for model calibration.

5. The authors extend their experimental panel with co treatment of GFP cells with nutlin in an attempt to obtain data for p53-Mdm2 protein dynamics. Unfortunately, the timescale of the model (0-60h) does not match the provided data in Figure 5C (30-60h). The most dynamic phase of protein regulation, according to the provided model fits (0-30h) is therefore not covered but would potentially provide valid insight into this dynamic phase. Data for nutlin treatment was provided for later timepoints, when the protein abundances in the model is already plateauing. Please include the data from the time series for time points 0-30h.

The reviewer correctly notices that, in the MDM2-inhibition experiment with Nutlin, the earliest measurement of p53 and MDM2 protein expression was at 24 hours and that subsequent dynamics were limited. We repeated the experiment to study the early dynamics and modified the manuscript accordingly.

6. The authors state in their discussion, p53 as well as Mdm2 mRNA levels have been shown to affect the protein levels of their respective counterpart. Therefore, we suggest adapting the model to the context of the well-known p53-mdm2 interaction, as we are confident that the body of p53-mdm2 literature potentially contains valuable additional information that could be included in the model. Potentially, this could strengthen the model in its predictive capacity. In fact, Mdm2 protein was previously described to directly affect p53 mRNA levels (DOI: 10.1038/ncb1770; DOI 10.1016/j.ccr.2011.11.016) which adds another layer of complexity on the p53-mdm2 regulatory mechanism. We suggest including these observations into the underlying equations in the model to better reflect known regulatory mechanisms. We therefore ask the authors again to consider adapting the model to include Mdm2 feedback into the p53 mRNA equation.

We agree with the reviewer that the model does not include possibly relevant feedback mechanisms, such as the MDM2 feedback on p53 mRNA levels. As is often the case with application of mathematical modeling to experimental data sets, data availability is limited, and models of high complexity are prone to overfitting. However, as indicated in our response to comment 4, we have added mRNA species for MDM2, p21 and BTG2 (note that we needed to fix a subset of parameters to avoid the discussed overfitting problem). Because the fit to our experimental protein expression data is already satisfactory (Fig. 3C), we argue that the current structure of the model is sufficient to simulate the protein dynamics, and any model extension that complicates the structure could lead to overfitting. Nevertheless, we also created an alternative model that includes some of the feedback mechanisms

suggested by the reviewer (Fig. S11). With this model, we were still not able to explain the negative correlation between MDM2 and TP53 mRNA. In addition, to improve the match between the predicted and measured MDM2 correlations at mRNA level, we altered the mathematical expression for MDM2 feedback on p53, but this did not improve the predictive capacity either. Both additional model versions are now briefly discussed in the final section of the Results, and model details are provided in the Supplement.

Minor concerns

1. Although the authors address the circumstance that p53 mRNA levels changes within the control conditions between 8h and 24h, we see this point critical. Fig 1B clearly indicates a time-dependent increase in p53 mRNA levels, which may be explained with difficulties in culturing PHHs and associated p53-mediated cell death (apoptosis) over time. How does a proliferating cell line (HepG2) and a quiescent state (PHHs) relate to each other? The authors partly addressed this difference with citing a paper in chapter 3, dealing with PHH stability in culture. However, p53 levels were not analysed in the cited paper. Given that p53 is involved in cell cycle regulation as well as cell death, we assume this reference as insufficient to explain p53 mRNA levels in PHHs. In consequence, we ask to authors to discuss this circumstance in more detail with additional literature.

As suggested by the reviewer, we have added additional discussion on PHH culturing and the temporal effect of culturing on mRNA expression (lines 598-615).

2. The authors state that the p53 protein levels peaked between 35 hours and 42 hours. Anyhow, they do not show data for untreated controls only for cisplatin concentration 1, 2.5, 5µM. Showing untreated samples would answer the question if there are basal changes in DNA damage with culturing time. This also leads to the question whether the confocal microscopy data (GFP-protein) is normalized to nuclei counts?

The raw data contains the integrated intensity of the GFP signal, i.e., the sum of all pixel values that belong to one cell, per identified cell nucleus or cytoplasm. To get population measurements per timepoint, we used the geometric mean of all cell measurements. Therefore, additional normalization to nuclei counts was not required. To correct for any effects in DMSO control condition we used background subtraction per time point, which also removes all dynamics from the DMSO control, i.e., all data points lie on the line y = 0. These details were already explained in the Methods, but in the revised manuscript we have now also briefly mentioned this in the Results section. Moreover, we now added a figure of the unnormalized data, including the dynamics at DMSO showing that there is minimal DNA damage-related stress in control conditions, as Fig. S6A.

3. The transcriptional activation of p21 and BTG2 by phosphorylated p53 was adapted to Mdm2 responsiveness in the model, which raises the question if this is a valid assumption, as the named targets react quite differently in the TempO-Seq analysis and experimental data about p53 phosphorylation is missing. We ask the authors to provide transcriptional activation data for p21 and BTG2 in either experimental setups or data from literature.

Protein p53 is known to transcriptionally induce the expression of MDM2, CDKN1A and BTG2 (https://doi.org/10.1016/j.gene.2017.01.018). To corroborate our assumption that

phosphorylated p53 is indeed increased upon cisplatin exposure and induces p21 protein expression, we included Methods and Results for Western blot experiments (Fig. S1B) in the revised manuscript.

4. We observe that, the initial mRNA expression analysis and the later described GFP intensities, i.e., protein levels are not very well linked together. We ask the authors to comment on whether they normalize protein GFP signals to the respective mRNA levels and how experiments from TempO-Seq and GFP-protein overexpression are represented in their model, besides setting p53 mRNA levels to 1.

We apologize for the prior unclarity with respect to normalization of the GFP signal and link between mRNA and protein expression in the model (see our replies to major point 4 and minor point 2). To prevent unclarity for future readers, in the revised manuscript we have extended our model with mRNA species for p21, BTG2 and MDM2. Note that we did not alter our strategy, i.e., we still only use the protein-GFP expression data to calibrate our model and subsequently derive predictions for mRNA expression from the model simulations.

5. Figure 5A shows the two best fits of parameters for the model that the authors found. However, we fail to comprehend why p53 protein levels decrease upon DNA damage, while phosphorylation of p53 and protein levels of all target genes increase. Given p53's role in DNA damage response, we would expect p53 protein levels to rise. Also, does the increase in p53-p take the decrease of total p53 into account in this model? Can this be normalized on total p53?

In control conditions, p53 is constantly produced and degraded. However, activation of p53 by phosphorylation stabilizes p53-p and leads to accumulation of p53-p in the cell. Even though p53 is still being produced, the conversion to p53-p is faster than the replenishment of p53, leading to a net decrease in unphosphorylated p53. Nevertheless, the reviewer is correct that the accumulation of p53-p leads to an overall increase of total p53 levels in the cell. To remove any unclarity about the model simulations for different p53 species and total p53, we adjusted the panels (now Fig. 4 and Fig. S7A), where we now show total p53 and its subspecies. As a side note, for our modified model with mRNA species for all p53 target genes we no longer found two fits of similar quality, so we removed this aspect from the manuscript.

6. The y-axis limits are quite different in the graphs describing the model fits (Fig 5) and nutlin experiments. E.g., in Fig 5C, D the y-axis should start at zero, otherwise differences appear greater than they actually are and consequently mislead the reader.

We adjusted the axis limits in the subfigures (now Fig. 4B-C) to start at 0.

7. Figure 6B shows that only the correlations of basal TP53 expression vs p53 protein levels after cisplatin treatment lines up between biological PHH samples and virtual samples. We ask how the authors ensure that their model is neither overfit nor underfit and discuss this accordingly.

With our bootstrap analysis, we show that there is quite some variation to be expected for correlations in similar sets of 50 PHH samples. Even though the medians of our model predictions and experimental mRNA data are not the same, the predictions do overlap with the regions of uncertainty for the PHH samples. Although we cannot know for sure whether our model is over- or underfitted, we have kept additions of model components for which we have no information at a minimum (see also our reply to major point 6) and fixed several parameters to 1, to circumvent identifiability issues. Our model likely needs additional factors to reproduce the negative TP53-MDM2 correlation, for which we currently have no data. To constrain the dynamics of such an extended model, additional experimental data on the extra components is necessary. We have clarified this in the revised manuscript (lines 630-643).

Reviewer #2

Heldring et al., use a mixture of in vitro experiments and in silico modeling to study how a well described feedback loop governing p53 regulation. They take this data and combine it with medium scale gene expression data from PHH samples. Though the authors have both RNA and protein datasets the lack of protein data for the PHH donors add complexity to their analysis. Overall, this paper both meets a critical need to develop approaches to leverage in vitro data to inform patient models and is insufficient to the task. As it stands the paper is only a weak fit for PLOS comp. Bio., however, plausible improvements might strengthen it considerably.

Major comments

(1) The computational model is thoughtfully designed, and well explained. Given the dominant role of transcription in regulation of downstream p53 targets, I would be interested to see if including mRNA species of p21/mdm2/btg2 would enable improved fits (obviously at a cost of higher complexity). In general, in addition to examining parameter sensitivity it would be useful if the authors looked at the robustness of their model to structural variation.

We agree with the reviewer that addition of mRNA species for MDM2, p21 and BTG2 will make the model better suited for our purpose. We added the species and modified our results and methods sections accordingly. As suggested by the reviewer, we in addition attempted altering of the model structure in various ways, i.e., by i) replacing the power 4 in the Hill equations with power 1, i.e., a Michaelis-Menten equation (Fig. S7), ii) changing the feedback of MDM2 on p53 with a Hill equation of power 4 (Fig. S11A), and iii) by using a different model structure for MDM2 and p53 mRNA and protein relations (Fig. S11B-D).

(2) The paper ends with a modeling approach to reconcile their patient data with a model of p53/mdm2 activity in response to cisplatin. Their virtual patient simulation is a reasonable approach. It would be very valuable to extend this analysis to its logical conclusion in giving treatment recommendations. If, for example, we assume high p21 levels are a ‘good’ clinical outcome what can we say about cisplatin dosing and the model parameters (and their steady state protein concentrations) that could achieve this outcome.

The suggestion of the reviewer touches upon a very interesting broad topic. Individual expression dynamics linked to DNA damage susceptibility and adverse outcomes could

provide valuable insights for the clinic. In our study, our main question is whether pathway dynamics in HepG2 cells can be translated to healthy PHHs. We show that relations between mRNA expression in model-based virtual samples are comparable to these relations in PHH samples. However, it is currently unclear what protein expression levels are associated with good prognostic outcome for a patient. For example, we do not know whether having a high cisplatin-induced p21 expression is indeed only associated with a good prognostic outcome for a patient, because high p21 expression is likely also associated with an increased probability for adverse effects in liver or kidney, which occurs in a large fraction of cisplatin-treated patients. Linking patient-specific expression to clinical outcome thus requires follow-up analysis, in which expression patterns are associated with a determined outcome and the generation of risk profiles. Because these variables are not covered in the TempO-Seq data set, our analysis does not allow us to give prognostic predictions on clinical outcome. Nevertheless, we have emphasized this in the discussion of our revised manuscript (lines 655-662).

(3) In figure 1, how can you hierarchically cluster based on one number (p53 or mdm2 expression)? Do the authors mean they arbitrarily grouped the cell samples? This seems essentially descriptive.

The TempO-Seq data contains multiple read-outs per PHH sample, i.e., there are both different doses and timepoints. This expression pattern was used to cluster the samples with hierarchical clustering. The dendrograms in the heatmaps (Figure 1 and S2) display the distance between the samples. This was already described in detail in the Methods, and in the revised version we have clarified this further in the Results section.

(4) Looking at 4 genes from a 1500 gene set to determine 'similarity' seems very odd (even if they are important genes). The authors should use a larger set (either the whole 1500) or subsets drawn from published 'gene-sets'.

We agree with the reviewer that the comparison of the response to cisplatin-induced DNA damage should also be studied in a broader context than merely selecting a few genes. However, a more extensive study of the entire gene set in PHH samples has already been performed in a different study that is currently under review and available on BioRxiv (https://doi.org/10.1101/2021.08.26.457742), which was possibly not entirely clear to the reviewer. As suggested by the reviewer, we added a comparison between the response to cisplatin in HepG2 and PHH using the entire gene set for which we used the PHH gene modules available in the TXG-MAPr (Fig. S1E). In the current manuscript, we further exploited the extensive gene expression data set by zooming into the DNA damage pathway to ask whether mechanistic insights in pathway regulation obtained for HepG2 cells can be translated to PHHs. We feel that our approach is a valuable addition on top of the broader analysis that has already been performed on the transcriptomics data. In the revised version of the manuscript, we have emphasized further that a more extensive bioinformatics analysis has been performed and that we here focus only on specific DNA damage components (lines 594-595).

(5) In figure 3 the authors seem to say they use p53 expression as proxy of cell survival/toxicity (341-343). As the authors later point out, this is a mis-reading of the

literature. Instead, the authors could use the expression of cell cycle gene expression (eg ccnd1, aurora A, mki67) as one proxy of cell health (although this may be complicated in the phh cells), or perhaps the activation of NFKB targets (triggered by cytoplasmic accumulation of DNA) such as TNFAIP3. More generally the authors should use their expression data more extensively to provide a more complex picture of the overall response of the phh cells to cisplatin treatment.

High concentrations of cisplatin are known to induce cell death due to extensive DNA damage. For both the TempO-Seq data and protein-GFP expression data, we aimed to determine at which concentrations cytotoxicity events were prevalent. Based on the enrichment for Gene Ontology (GO) terms associated to cell death, we found that genes related to cytotoxicity were upregulated at 10 µM cisplatin or higher (Fig. S4). Similarly, propidium iodide (PI) and annexin-V (AnV) staining in HepG2 cells showed increased PI and AnV positive cells at 10 µM cisplatin or higher (Fig. S6). As suggested by the reviewer, we now provide further support to our assumption by basing cell health proxies also on enrichment of cell cycle-associated GO terms. We added the results to Fig. S4 and adjusted the manuscript to clarify our methods to determine cytotoxicity (lines 178-181 in the Methods and 404-408 in the Results).

Minor comments

(1) The paper figures are well drawn, but could be compacted considerably, the core point of the paper draws very little on data from figures 1-3 (for example figure 3 is basically a negative result) and this part could be condensed.

We agree with the reviewer observation that Fig. 1-3 is not the core point of our paper. Therefore, we have moved subfigure 3A and 3B to the supplementary material and condensed Figure 1-3 into two figures.

(2) Overall in the paper text there is some confusion between RNA and protein measurements and what each imply (not on the authors part I believe, but given their different measurements its important to be very specific in the text to avoid mis-reading). This is critical to this circuit so the authors should clarify this in each section.

We apologize that, despite our efforts to be precise in our wording, the distinction between mRNA and protein was not clear. With the addition of mRNA species to the model and consequent textual adjustments, we hope to have resolved this issue.
